# MicroRNA-200b-3p restrains gastric cancer cell proliferation, migration, and invasion via C-X-C motif chemokine ligand 12/CXC chemokine receptor 7 axis

**DOI:** 10.1080/21655979.2022.2034585

**Published:** 2022-02-28

**Authors:** Dinuo Li, Qiang Li

**Affiliations:** a Department of General Gastropathy, The First Affiliated Hospital of Jinzhou Medical University, Jinzhou, China; b Department of Gastrosurgery, The First Affiliated Hospital of Jinzhou Medical University, Jinzhou, China

**Keywords:** miR-200b-3p, CXCL12/CXCR7, GC, proliferation, migration and invasion

## Abstract

This study was conducted to investigate the impact of microRNA (miR)-200b-3p on viability, migration, and invasion of gastric cancer (GC) cells and its mechanism. Quantitative real-time PCR (qRT-PCR) was conducted to measure miR-200b-3p expression in GC tissues and cells; besides, the relationship between miR-200b-3p expression and overall survival time (OS) was analyzed with OncomiR database; cell counting kit-8 (CCK-8), colony formation assay, flow cytometry, scratch healing assay, and Transwell assay were performed to detect the proliferation, cell cycle progression, migration, and invasion of GC cells; a lung metastasis model in nude mice was used to examine the effect of miR-200b-3p on the metastasis of GC cells *in vivo*; the interplay between miR-200b-3p and C-X-C motif chemokine ligand 12 (CXCL12) mRNA 3’ UTR was predicted by bioinformatics and verified with a dual-luciferase reporter gene assay; besides, the expression of CXCL12 and CXC chemokine receptor 7 (CXCR7) was probed by Western blot. It was found that miR-200b-3p expression was down-regulated in GC tissues, which was remarkably associated with the lymph node metastasis and decrease of differentiation of GC; transfection with miR-200b-3p mimics restrained the growth, migration, and invasion of GC cells *in vitro*, induced cell cycle arrest, and inhibited CXCL12 and CXCR7 expression levels; transfection of miR-200b-3p inhibitors worked oppositely *in vitro* and promoted lung metastasis *in vivo*. CXCL12 was confirmed as the downstream target of miR-200b-3p and was negatively modulated by miR-200b-3p. In conclusion, miR-200b-3p inhibited GC progression via regulating CXCL12/CXCR7 axis.

## GRAPHICAL ABSTRACT

**Figure:**
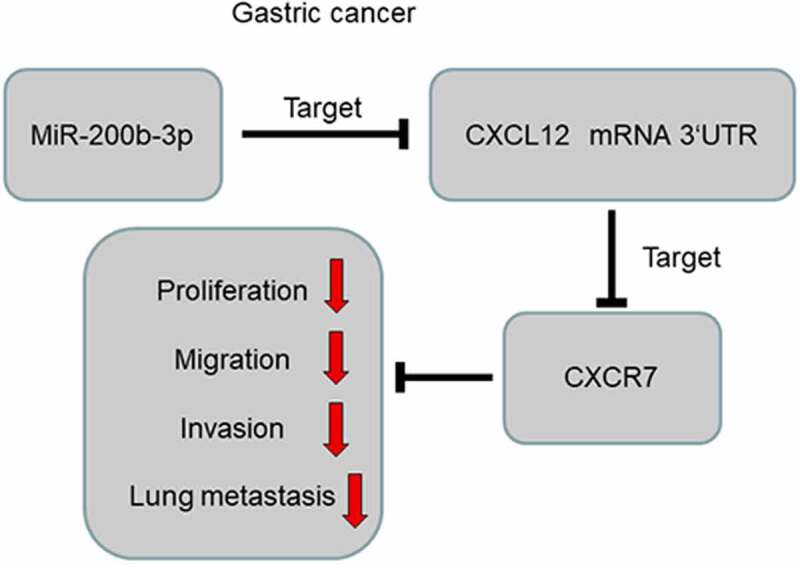

## 1 Introduction

Gastric cancer (GC) is one of the most common human malignancies, which has high morbidity and mortality in the globe and is the third driving cause of cancer-related death [1]. Currently, the main treatments for GC in clinical practice include surgical treatment, radiation therapy, chemotherapy, and molecular targeted therapy [2]. Most of the sufferers are diagnosed in the advanced stage, the possibility of radical surgery is lost; additionally, GC cells are prone to show resistance to radiotherapy and chemotherapy, which seriously limits the therapeutic effect [3,4]. It is vital to elucidate the mechanism of GC pathogenesis and find new diagnostic biomarkers and therapeutic targets.

MicroRNA (miRNA), known as a single-stranded non-coding RNA of 18–25*nt* in length, silences the expression of target genes mainly by completely or incompletely complementary pairing with the 3′-untranslated region (3′-UTR) of mRNAs, causing degradation or translational inhibition of mRNAs. Reportedly, miRNAs are pivotal in the progression of diverse cancers [5,6]. As a vital player of the miRNA family, miR-200b-3p has important biological functions in the tumor progression. For example, miR-200b-3p inhibits the growth and migration, and promotes the chemosensitivity of colorectal cancer cells via restraining TUBB3 expression [7]; miR-200b-3p directly targets SMAD2 to promote proliferation, invasion, and epithelial–mesenchymal transition (EMT) of melanoma cells [8]. However, how miR-200b-3p works in GC is not clear.

Abnormal activation of the C-X-C motif chemokine ligand 12 (CXCL12)/CXC chemokine receptor 7 (CXCR7) axis causes abnormal cell proliferation and differentiation and contributes to tumorigenesis [9,10]. Specifically, CXCL12/CXCR7 axis is implicated in promoting GC cell proliferation, associated with lymph node and liver metastasis [10]. In the present work, bioinformatics analysis showed that miR-200b-3p could probably directly target CXCL12 3ʹUTR. We hypothesized that miR-200b-3p had the potential to be the diagnostic biomarker and therapeutic target for GC. We investigated the biological function and mechanism of miR-200b-3p in GC progression. Herein, we report that miR-200b-3p inhibits GC cell growth, migration and invasion through targeting the CXCL12/CXCR7 axis.

## 2. Materials and methods

### 2.1. Collection of tissue samples

Fifty-nine GC tissue samples and their adjacent normal samples used in this study were obtained from the surgically resected tumor tissues and adjacent non-cancerous tissues in The First Affiliated Hospital of Jinzhou Medical University, and the samples were subsequently frozen in liquid nitrogen within 30 minutes. All of the patients were diagnosed as GC by clinical symptoms, imaging, and pathological examination, and all patients had no other tumors (among the 59 cases, 18 of the 59 GC cases were the intestinal type, 25 were the diffuse type and 16 were the mixed type; 29 cases in TNM stage I–II, 30 cases in TNM stage IIII-IV). All subjects did not receive chemotherapy, radiotherapy, and other anti-cancer treatment before the surgery. All of the subjects signed informed consent before the surgery. This work was accomplished under the approval and guidance of the Ethics Committee of the First Affiliated Hospital of Jinzhou Medical University (ethical number: 201705A13).

### 2.2. *Cell culture* [10]

Gastric epithelial cells GES-1 were available from China Center for Type Culture Collection (CCTCC, Wuhan, China), and GC cell lines (AGS, SNU-16, SNU-1, and NCI-N87) were from American Type Culture Collection (ATCC) (Manassas, VA, USA). The cells were cultured in Dulbecco’s modified Eagle medium (DMEM) (Invitrogen, Carlsbad, CA, USA) with 10% fetal bovine serum (Invitrogen, Carlsbad, CA, USA) +100 U/ml penicillin (Invitrogen, Carlsbad, CA, USA) +100 μg/ml streptomycin (Invitrogen, Carlsbad, CA, USA) at 37°C, in 5% CO_2_ and 95% humidity. When the cells were in the logarithmic growth stage, they were harvested for the subsequent experiments.

### 2.3. *Cell transfection* [11]

AGS and SNU-1 cells were inoculated into 60 mm dish (1 × 10^6^ cells per dish) and subsequently cultured at 37°C in 5% CO_2_ for 24 h, and then the transfection was performed according to the manufacturer’s instruction of Lipofectamine® 2000 kit (Invitrogen, Carlsbad, CA, USA). The mimics NC, inhibitors NC, miR-200b-3p mimics (miR mimics), miR-200b-3p inhibitors (miR inhibitors), CXCL12 overexpression plasmid (CXCL12) and small interference RNA targeting CXCL12 (si-CXCL12) were all available from RiboBio Co, Ltd. (Guangzhou, China). In the transfection, the concentration of the mimics and inhibitors was 50 nM. 24 h after the transfection, the transfection efficiency was validated by quantitative real-time PCR (qRT-PCR).

### 2.4. *qRT-PCR* [12]

Total RNA was extracted from tissues or cells by TRIzol reagent (Invitrogen, Shanghai, China). RNA was reversely transcribed into cDNA by a Reverse Transcription Kit (Takara, Dalian, China). The real-time PCR analysis was performed in the ABI7500 real-time PCR system (Applied Biosystems, San Francisco, CA, USA) with a Power SYBR Green PCR Master Mix kit (Takara, Dalian, China). Specifically, the relative expression of miR-200b-3p and CXCL12 was examined by 2^−ΔΔCt^ with β-actin and U6 as the internal reference. Primer sequence is as follows: miR-200b-3p forward: 5′-GCTGCTGAATTCCATCTAATTTCCAAAAG-3′, reverse: 5′-TATTATGGATCCGCCCCCAGGGCAATGGG-3′;

CXCL12 forward: 5′-ATGCCCATGCCGATTCTT-3′, reverse: 5′-GCCGGGCTACAATCTGAAGG-3′;

U6 forward: 5′-TGCGGGTGCTCGCTTCGGCAGC-3′, reverse: 5′-CCAGTGCAGGGTCCGAGGT-3′;

β-actin forward: 5′-CCAGTGCAGGGTCCGAGGT-3′, reverse: 5′-ACTCCTGCTTG CTGATCCAC-3′.

### 2.5. *Cell counting kit-8 (CCK-8) assay* [13]

The GC cells were prepared into single-cell suspension. Next, the GC cells were inoculated into 96-well plates at 1000 cells/well. Next day, 90 μL of medium and 10 μL of CCK-8 solution (Dojindo Molecular Technologies, Japan) were added into each well, and the cells were cultured for 2 h. Next, the absorbance (optical density, OD) value was examined and recorded with a microplate reader at the wavelength of 450 nm every 24 h for consecutive 4 days. The growth curve was drawn, with time as abscissa and OD_450nm_ value as ordinate.

### 2.6. *Colony formation assay* [14]

Transfected GC cells were seeded into 6-well plates (1 × 10^3^/well). After 2 week-culture, the medium was discarded, and the cells were fixed with 100% methanol and then stained with 0.1% crystal violet solution for 15 min. Finally, the colonies were washed, and the number of colonies in each group was counted and recorded.

### 2.7. *Scratch healing assay* [15]

Transfected AGS and SNU-1 cells were subsequently inoculated in a 6-well plate at 1 × 10^6^ cells /well, and 2 ml of complete medium was loaded. When the cell confluence reached 80%–90%, the cells were scratched vertically with a pipette and washed twice with phosphate buffer saline (PBS). The scratches were observed and photographed by an inverted microscope. Next, the cells were cultured with serum-free medium. After 24 h, the scratch was observed again. Scratch healing rate (%) = (scratch width of 0 h-scratch width of 24 h)/scratch width of 0 h × 100%.

### 2.8. *Transwell assay* [16]

Migration assay: AGS cells and SNU-1 cells in logarithmic phase of growth were resuspended in serum-free medium, and inoculated into the upper compartment of transwell chamber (Corning incorporated, Corning, NY, USA) (1 × 10^4^ cells/well). Subsequently, 500 μL of complete medium with 10% serum was added into the lower compartment. The cells were then cultured for 24 h. The cells which failed to migrate were gently wiped off with a cotton swab. The migrated cells were fixed with 4% paraformaldehyde for 15 min and then stained with 0.1% crystal violet for 10 min. After the cells were washed by tap water, five visual fields were selected randomly under a microscope and the stained cells were counted. In the invasion assay, the upper chamber of Transwell system was pre-covered with a layer of Matrigel, and the remaining procedures of invasion assay were the same as the migration assay.

### 2.9. *Flow cytometry* [17]

The cell cycle was analyzed by flow cytometry. The cells were rinsed twice with PBS, fixed with 70% ethanol, and stored overnight at 4°C. On the second day, the cells were washed in PBS, with the density adjusted to 1 × 10^6^ cells /mL. Subsequently, the cells were mixed with the propidium iodide staining solution until the final concentration reached 0.05 mg/mL, and then stained at 4°C for 30 min. Finally, the cell cycle of the GC cells was determined by a flow cytometer.

### 2.10 *Lung metastasis assay in vivo* [18]

Male BALB/c nude mice (9 weeks old) were obtained from the Experimental Animal Center of Jinzhou Medical University (Jinzhou, China). The mice were randomly divided into two groups (inhibitors NC group and miR inhibitors group). Then, SNU-1 cells were inoculated into nude mice (6 mice per group) through the tail vein (1 × 10^7^ cells per mouse). After 2 weeks, the mice were euthanized, and the lungs were obtained, fixed with formalin, embedded in paraffin, and then serially sectioned. After that, hematoxylin and eosin (H&E) staining was performed, and the lung metastatic nodules were examined under a microscope by a pathologist.

### 2.11. *Dual luciferase reporter gene assay* [19]

The binding site between miR-200b-3p and CXCL12 mRNA 3ʹUTR was predicted by StarBase database, and wild-type (WT) CXCL12 sequence was amplified and inserted into pmirGLO dual-luciferase miRNA target expression vectors (Promega Corp., Madison, WI, USA) to construct the reporter vector pmirGLO-CXCL12-WT (WT CXCL12). A GeneArt™ Site-Directed Mutagenesis PLUS System (Thermo Fisher Scientific, Inc., MA, USA) was used to mutate the binding site miR-200b-3p and CXCL12 mRNA 3ʹUTR. Next, the mutant (MUT) sequence was inserted into the pmirGLO vector to construct reporter vector pmirGLO-CXCL12-MUT (MUT CXCL12). The above vectors were co-transfected with mimics NC, miR-200b-3p mimics and inhibitors NC, miR-200b-3p inhibitors into AGS and SNU-1 cells, respectively. 48 h later, the luciferase activity in each group was determined. The ratio of the luminescence intensity of Renilla luciferase to that of firefly luciferase was used to reflect the binding intensity of miR-200b-3p to CXCL12 3ʹUTR.

### 2.12. *Western blot* [20]

RIPA lysis buffer containing protease inhibitor (Beyotime Biotechnology, Shanghai, China) was used to lyse the GC cells at 4°C, and centrifuged at 12,000 r/min for 5 min, with the supernatant collected as the whole protein extract. The BCA protein quantification kit (Beyotime Biotechnology, Shanghai, China) was employed to determine the protein concentration. Next, the protein samples were mixed with loading buffer, and the proteins were denatured in boiling water for 5 min. The protein samples were dissolved via sodium dodecyl sulfate polyacrylamide gel electrophoresis, then transferred to polyvinylidene fluoride (PVDF) membrane, which was then blocked by 5% skimmed milk for 2 h at ambient temperature. Rabbit anti-CXCL12 antibody (cell signaling technology, 3530, 1:1000) and rabbit anti-β-actin antibody (Abcam, ab8227, 1:1000) were loaded and the membrane was incubated overnight at 4°C. Next, the membrane was washed with tris buffered saline tween (TBST), and then incubated with goat anti-rabbit IgG H&L (HRP) (Abcam, ab205718,1:2000) for 1 h. After the PVDF membrane was washed in TBST, the protein bands were developed by a high-sensitivity ECL chemiluminescence kit (Beyotime, Shanghai, China).

### 2.13. *Statistical analysis* [21]

All the experiments were conducted three times. SPSS 22.0 statistical software (SPSS Inc., Chicago, IL, USA) was used to analyze the experimental data, with data expressed as mean ± standard deviation. *t*-test was adopted for comparisons between two groups. One-way analysis of variance was used for comparisons among multiple groups and Tukeys multiple comparisons test was used to define differences between groups. Pearson correlation coefficients were used to examine the correlation between two normally distributed variables. The survival analysis was conducted with Kaplan–Meier method using OncomiR database (http://www.oncomir.org). The enumeration data were expressed in a contingency table, and χ2 test was executed to analyze the differences between the two sets. Statistically, *P* < 0.05 is meaningful.

## 3. Results

We hypothesized that miR-200b-3p could inhibit the progression of GC. Gain-of-function and loss-of-function models were established, and it was demonstrated that miR-200b-3p could regulate the malignant biological behaviors of GC cells *in vitro* and *in vivo*. Additionally, it was demonstrated that miR-200b-3p directly targeted CXCL12/CXCR7 pathway, and the overexpression of CXCL12 reversed the inhibiting effects of miR-200b-3p on proliferation, migration, and invasion of NSCLC cells.

### 3.1. MiR-200b-3p expression characteristics in GC tissues and cells

**Figure 1.**
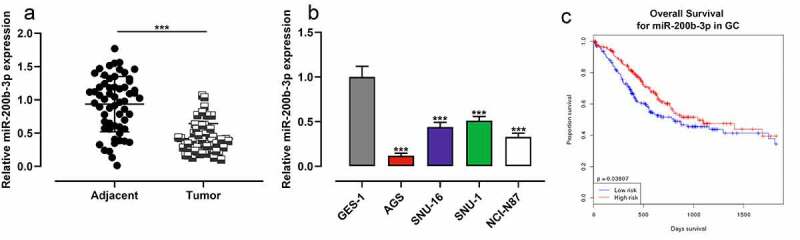
MiR-200b-3p is down-regulated in GC, and it is a potential prognostic factor.

To study miR-200b-3p’s expression characteristics in GC, qRT-PCR was performed and it was revealed that miR-200b-3p expression in GC was markedly inhibited compared with that in adjacent tissues (Figure 1(a)); as against GES-1 cells, miR-200b-3p expression in GC cell lines (AGS, SNU-16, SNU-1, and NCI-N87) was significantly down-regulated (Figure 1(b)). OncomiR database showed that low expression of miR-200b-3p was associated with the short overall survival (OS) of GC patients (Figure 1(c)). After the patients were classified into high and low miR-200b-3p expression groups based on the average value of miR-200b-3p expression, chi-square test suggested that the low miR-200b-3p was closely associated with the lymph node metastasis and poor differentiation of GC tissues of GC patients (Table 1).

**Table 1.** The correlation between miR-200b-3p expression and its clinicopathological features in GC

Pathological	Numbers (n = 59)	miR-200b-3p expression High(n = 30) Low(n = 29)		χ^2^	*p-*Value
Age (years)				0.1360	0.712
<55	38	20	18		
≥55	21	10	11		
Tumor size (cm)				0.407	0.524
<5	35	19	16		
≥5	24	11	13		
Degree of differentiation				4.984	0.026*
Low, medium	32	12	20		
High	27	18	9		
Lymph node metastasis				4.002	0.045*
No	38	23	15		
Yes	21	7	14		
TNM stage				1.379	0.240
I–II	29	17	12		
III–IV	30	13	17		

*P < 0.05.

A. MiR-200b-3p expression in GC tissues and normal tissues adjacent to cancer was detected byqRT-PCR.

B. MiR-200b-3p expression in GES-1 cells and GC cell lines were detected by qRT-PCR.

C. The association between the prognosis of GC patients and the expression of miR-200b-3p was analyzed using OncomiR database.

*** *P* < 0.001.

### 3.2. Impacts of miR-200b-3p on growth and cell cycle progression of GC cells

**Figure 2.**
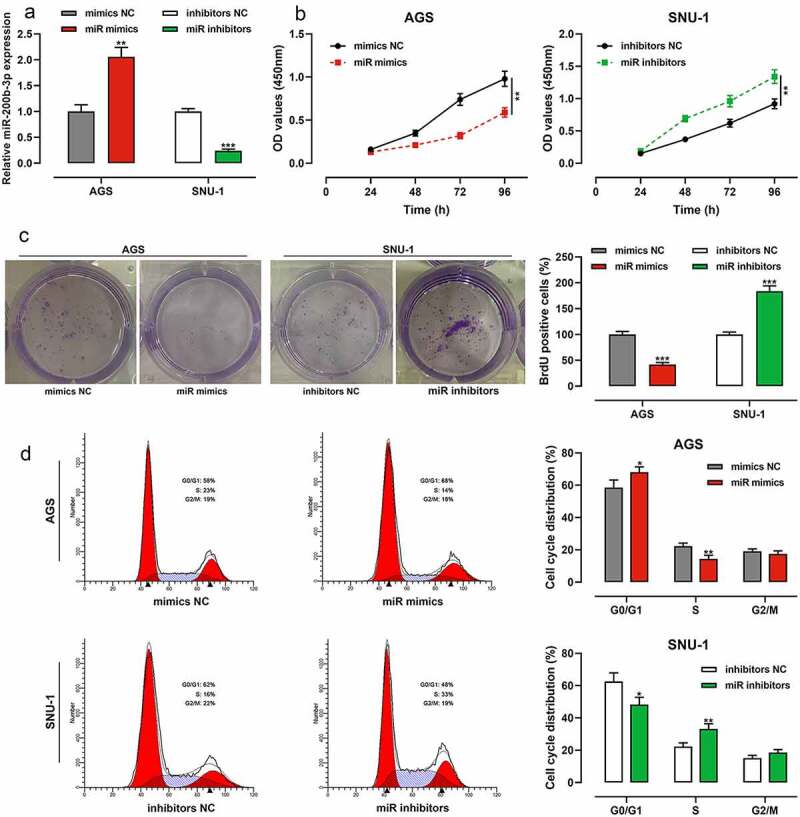
Effects of miR-200b-3p on growth and cell cycle of GC cells.

Among GC cells, the expression of miR-200b-3p was lowest in AGS cells, so AGS cells were selected for subsequent miR-200b-3p overexpression experiments; the expression of miR-200b-3p was highest in SNU-1 cells, so SNU-1 cells were selected for subsequent miR-200b-3p inhibition experiments. qRT-PCR suggested that the transfection was successful (Figure 2(a)). CCK-8 assay showed that as against the control group, miR-200b-3p overexpression markedly inhibited the growth of AGS cells, while inhibition of miR-200b-3p promoted that of SNU-1 cells (Figure 2(b)). Notably, colony formation assay suggested that compared with the control group, miR-200b-3p overexpression markedly inhibited the colony forming ability of AGS cells, while inhibition of miR-200b-3p significantly promoted that of SNU-1 cells (Figure 2(c)). In addition, flow cytometry assay showed that in comparison with the control group, up-regulation of miR-200b-3p significantly induced G0/G1 cycle arrest of AGS cells, while miR-200b-3p inhibition promoted the cell cycle progression of SNU-1 cells (Figure 2(d)).

A. The transfection efficiency of miR-200b-3p mimics and inhibitors was detected by qRT-PCR.

B. The effects of miR-200b-3p mimics and inhibitors on the viability of AGS and SNU-1 cells were under the detection of CCK-8 assay.

C. The effects of miR-200b-3p mimics and inhibitors on the proliferation of AGS and SNU-1 cells were detected by colony formation experiment.

D. The effects of miR-200b-3p mimics and inhibitors on AGS and SNU-1 cell cycle were detected by flow cytometry.

* *P* < 0.05, ** *P* < 0.01, and *** *P* < 0.001.

### 3.3. Influence of miR-200b-3p on migration and invasion of GC cells

**Figure 3.**
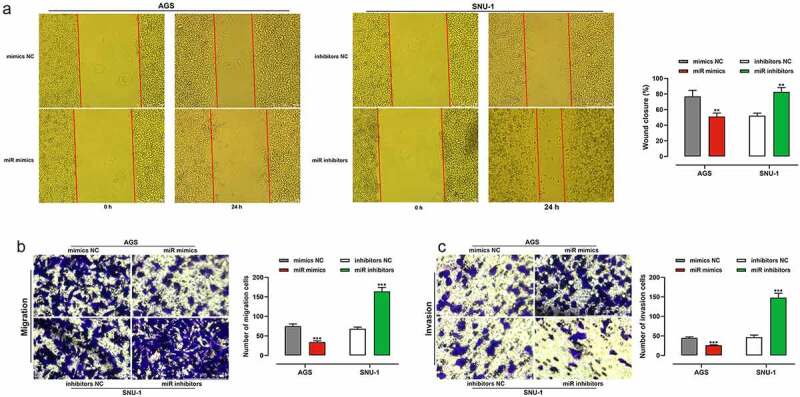
Effects of miR-200b-3p on migration and invasion of GC cells.

To clarify the impacts of miR-200b-3p on migration and invasion of GC cells, we adopted scratch healing assays and transwell assays and proved that transfection of miR-200b-3p mimics markedly restrained the migration and invasion of AGS cells; however, transfection of miR-200b-3p inhibitors dramatically enhanced the capability of migration and invasion of SNU-1 cells (Figure 3(a-c)). To further consolidate that miR-200b-3p suppressed BC progression, lung metastasis assay in nude mice was performed, and it indicated that inhibition of miR-200b-3p promoted lung metastasis *in vivo* (Supplementary Figure S1).

A. The effects of miR-200b-3p mimics and miR-200b-3p inhibitors on the migration of AGS and SNU-1 cells were examined by scratch healing assay.

B, C. Transwell assay was used to investigate the regulatory effects of miR-200b-3p mimics and inhibitors on the migration and invasion of AGS and SNU-1 cells.

** *P* < 0.01 and *** *P* < 0.001.

### 3.4. MiR-200b-3p targets CXCL12 in GC cells

**Figure 4.**
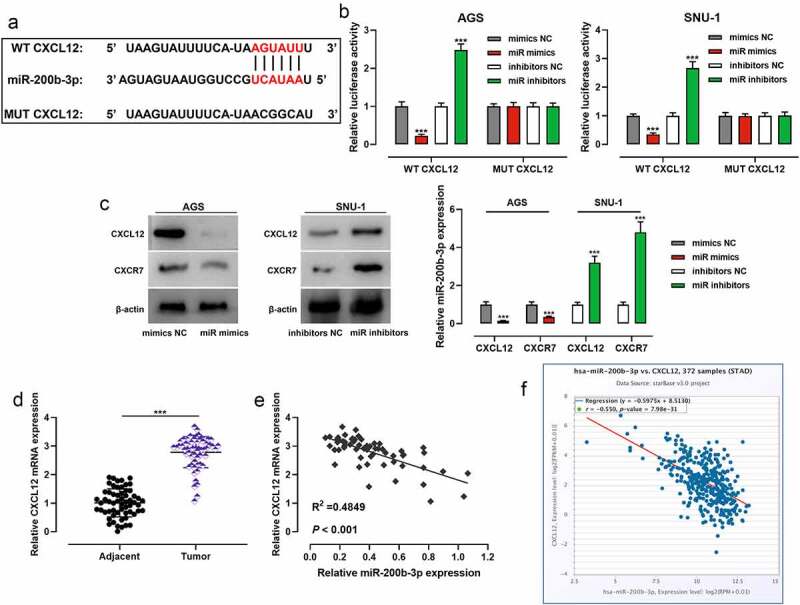
MiR-200b-3p targets CXCL12 in GC cells.

To decipher the downstream mechanism of miR-200b-3p, StarBase database was searched, and it showed that CXCL12 may be one of the functional targets of miR-200b-3p (Figure 4(a)). To confirm the targeting relationship between miR-200b-3p and CXCL12, dual-luciferase reporter gene assay was performed, and it showed that transfection of miR-200b-3p mimics inhibited the luciferase activity of WT CXCL12, while transfection of miR-200b-3p inhibitors promoted that of WT CXCL12; transfection of miR-200b-3p mimics or inhibitors exerted no obvious effect on that of MUT CXCL12 (Figure 4(b)). Western blot showed that transfection of miR-200b-3p mimics inhibited CXCL12 and CXCR7 protein expression levels, and transfection of miR-200b-3p inhibitors promoted their protein expression levels (Figure 4(c)). qRT-PCR suggested that CXCL12 mRNA expression was remarkably higher in GC tissues than that in adjacent tissues (Figure 4(d)), and CXCL12 mRNA expression was negatively correlated with miR-200b-3p expression (Figure 4(e)). Furthermore, StarBase database also showed that there was a negative correlation between them (Figure 4(f)).

A. The binding site between miR-200b-3p and CXCL12 3ʹUTR was predicated by StarBase database.

B. The effects of miR-200b-3p mimics and inhibitors on luciferase activity of WT CXCL12 and MUT CXCL12 in AGS and SNU-1 cells were detected by dual-luciferase reporter gene assay.

C. Western blot was used to examine the effect of miR-200b-3p mimics and miR-200b-3p inhibitors on the protein expression levels of CXCL12 and CXCR7 in AGS and SNU-1 cells.

D. CXCL12 mRNA expression in GC and adjacent tissues was detected by qRT-PCR.

E, F. Pearson correlation analysis and StarBase database confirmed the correlation between miR-200b-3p expression and CXCL12 expression.

*** *P* < 0.001.

### 3.5. MiR-200b-3p influenced GC cells through repressing CXCL12

**Figure 5.**
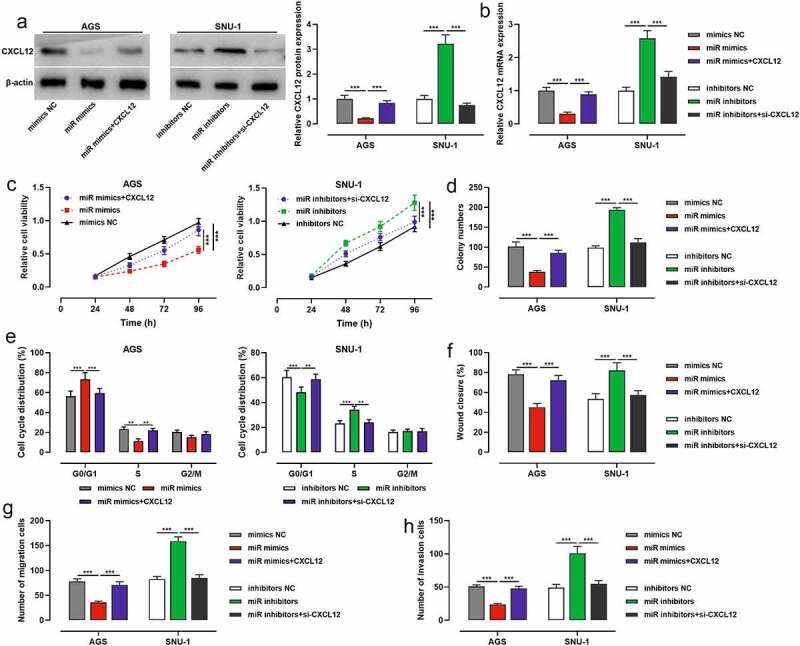
Effects of miR-200b-3p and CXCL12 on proliferation, migration and invasion and cell cycle of GC cells.

To further explore whether miR-200b-3p exerted its biological functions through CXCL12, we co-transfected miR-200b-3p mimics and CXCL12 overexpression plasmids into AGS cells, and miR-200b-3p inhibitors and si-CXCL12 into SNU-1 cells, with Western blot and qRT-PCR showing it a success (Figure 5(a,b)). CCK-8 assay, colony formation assay, flow cytometry assay, scratch healing assay and Transwell assay highlighted that transfection of miR-200b-3p mimics restrained AGS cell viability, growth, migration, and invasion, and arrested the cell cycle, while CXCL12 overexpression reversed the above effects (Figure 5(c-h)); transfection of miR-200b-3p inhibitors significantly promoted the malignant biological behaviors of SNU-1 cells, and transfection of si-CXCL12 weakened the above effects (Figure 5(c-h)).

A, B. The transfection efficiency of miR-200b-3p mimics and CXCL12 overexpression plasmid, and the transfection efficiency of miR-200b-3p inhibitors and si-CXCL12 were examined by Western blot and qRT-PCR.

C. The regulatory effects of miR-200b-3p and CXCL12 on the viability of AGS and SNU-1 cells were detected by CCK-8 assay.

D. The regulatory effects of miR-200b-3p and CXCL12 on the proliferation of AGS and SNU-1 cells were detected by colony formation assay,

E. The regulatory effects of miR-200b-3p and CXCL12 on AGS and SNU-1 cell cycle were evaluated by flow cytometry.

F. The regulatory effects of miR-200b-3p and CXCL12 on the migration of AGS and SNU-1 cells were examined by scratch healing assay.

G, H. Transwell assay was used to detect the regulatory effects of miR-200b-3p and CXCL12 on the migration and invasion of AGS and SNU-1 cells.

** *P* < 0.01 and *** *P* < 0.001.

## 4. Discussion

MiRNAs are vital regulators in cancer biology, and they are implicated in modulating diverse biological processes, including cell growth, survival, differentiation, metabolism, inflammation, invasion and angiogenesis via modulating the expression of target genes [22,23]. In GC, miRNAs can serve as cancer-promoting or cancer-inhibiting factors [,^24^]. MiR-200b-3p is involved in regulating the progression of various tumors, for instance, miR-200b-3p restrains colorectal cancer cell growth and induces apoptosis by blocking the Wnt/β-catenin pathway through targeted inhibition of Wnt1 expression [25]; miR-200b-3p restrains the MAPK signaling pathway by repressing HMGB3 expression, ultimately restraining the malignant biological behaviors of glioblastoma multiforme cells [26]; miR-200b-3p represses the viability, migration, and EMT of pancreatic cancer cells via suppressing ZEB1 expression [27]; miR-200b-3p can promote the growth, migration, and invasion of lung adenocarcinoma cells via targeting and inhibiting ABCA1 expression [28]. Here we report that miR-200b-3p expression is markedly down-regulated in GC tissues and cells and correlated with poor prognosis of patients, and miR-200b-3p can repress the viability, aggressiveness and cell cycle progression of GC cells. These data suggest that miR-200b-3p is a tumor suppressor in GC.

Chemokines are a class of small-molecule cytokines with a molecular weight of roughly 8–10 kD [29,30]. Chemokine receptors are a superfamily of G-protein-coupled receptors containing seven-transmembrane structures, which are divided into four major categories: CR, CCR, CXCR, and CX3CR [31]. By specifically recognizing the corresponding chemokines, chemokine receptors are vital in mediating various physiological and pathological processes, including leukocyte migration, embryonic development, inflammatory responses, angiogenesis, and tumorigenesis [32]. CXCL12 belongs to the CXC chemokine subfamily, which binds to CXCR7 [33]. When CXCR7 binds to its ligand CXCL12, it can activate inflammatory signaling pathways such as NF-κB, thus regulating angiogenesis, inflammation, and stress response [34]. For example, in inflammatory bowel disease (IBD), CXCL12 expression is upregulated compared with normal tissues, and CXCR7 expression is raised in human peripheral blood T cells (PBTs) from IBD patients compared with PBTs of healthy subjects, and aberrantly activated CXCR7 can aggravate intestinal inflammation in IBD patients [35–37]. Importantly, CXCL12/CXCR7 pathway is also involved in many kinds of tumors including GC. For example, CXCL12/CXCR7 can activate Rho/ROCK pathway and promote the migration and invasion of pancreatic cancer cells [38]; CXCL12/CXCR7 promotes the malignancy of renal cancer cells by activating mTOR signaling pathway [39]. In GC, it is reported that high expression of CXCL12/CXCR7 is markedly associated with advanced tumor stage, lymph node metastasis, and liver metastasis, and aberrantly activated CXCL12/CXCR7 promotes the malignant biological behaviors of GC cells [10]. Consistently, some other studies report that high expression of CXCR7 is associated with unfavorable pathological characteristics of GC patients, and CXCR7 promotes the growth, migration, invasion, adhesion, and angiogenesis of GC cells [40,41]. The present work report that CXCL12 is a downstream target of miR-200b-3p, which directly targets and negatively modulates CXCL12 expression. Interestingly, we also observed that CXCR7 expression was suppressed by miR-200b-3p, and the molecular mechanism remains to be explored in the following studies.

The current research has some limitations. First, the mechanism by which miR-200b-3p affects the pathogenesis of GC needs to be further elucidated in the future, and miR-200b-3p may exert its tumor-suppressive effect via other downstream genes. Additionally, considering that CXCL12/CXCR7 is involved in modulating angiogenesis, it is also interesting to explore whether miR-200b-3p has the potential to repress angiogenesis of GC tissues.

## 5 Conclusion

Collectively, we reported that miR-200b-3p expression was down-regulated in GC, which implied the poor prognosis of the patients. Functionally and mechanistically, miR-200b-3p is a tumor suppressor in GC by targeting CXCL12/CXCR7 pathway. This work is helpful to explain the molecular mechanism of GC progression, and provides theoretical basis for developing novel diagnosis biomarker and therapy target of GC.

## Supplementary Material

Supplemental Material

## Data Availability

The data used to support the findings of this study are available from the corresponding author upon request.

## Highlights

- MiR-200b-3p expression was significantly down-regulated in GC tissues and cells.
- MiR-200b-3p inhibition promoted the growth, migration, invasion, and cell cycle progression of GC cells.
- MiR-200b-3p negatively regulated CXCL12 and CXCR7 expression levels in GC cells.
