# Supplementary material for: MicroRNA-200b-3p restrains gastric cancer cell proliferation, migration, and invasion via C-X-C motif chemokine ligand 12/CXC chemokine receptor 7 axis
Source: Bioengineered. 2022 Feb 28;13(3):6509–20. doi: 10.1080/21655979.2022.2034585 (PMC8974025; doi:10.1080/21655979.2022.2034585)
Supplement: Supplemental Material [file KBIE_A_2034585_SM7530.zip › supplementary/Supplementary Materialsclean.docx]

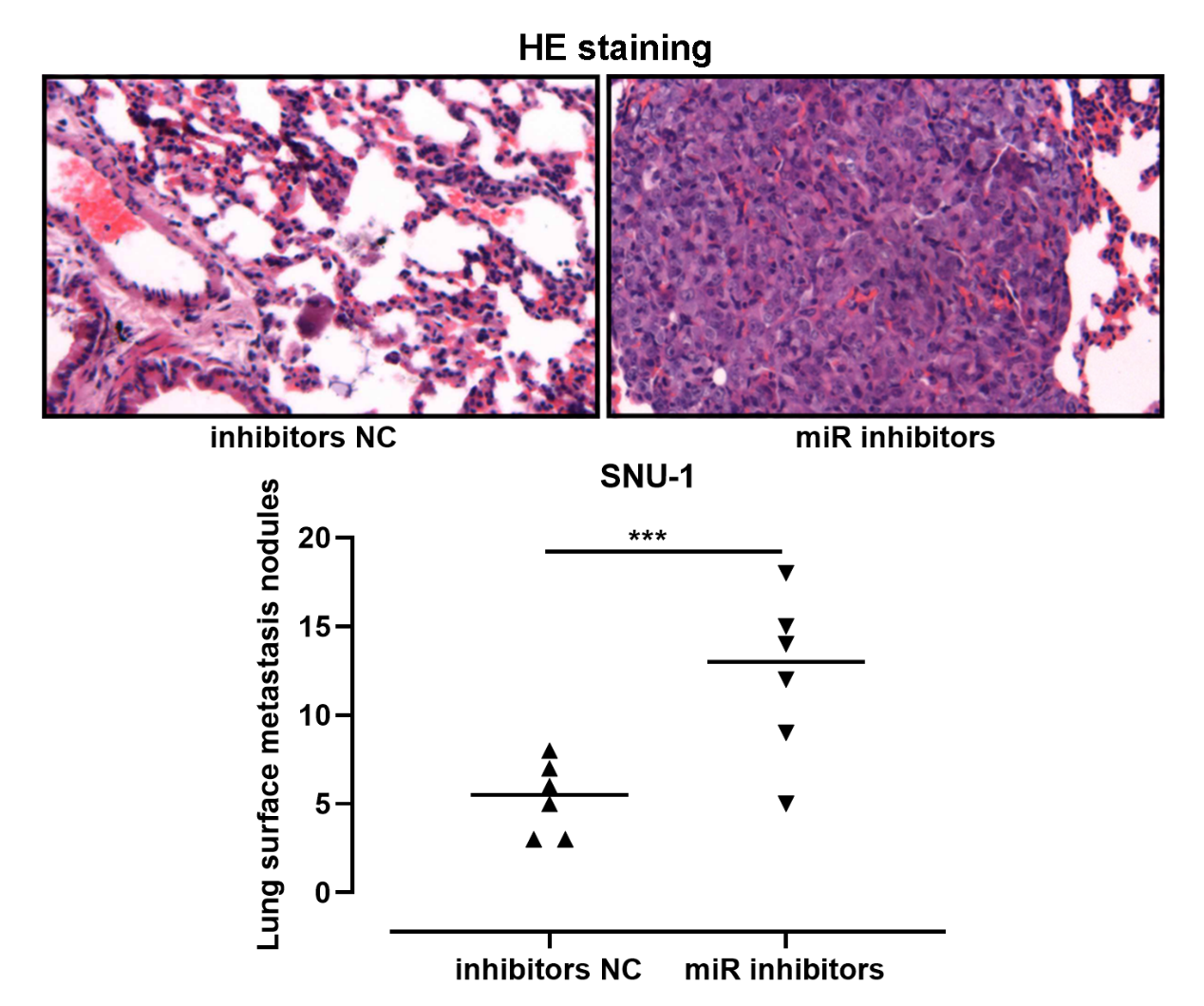


***Supplementary Figure 1***

Lung metastasis of GC cells *in vivo* was evaluated after the nude mice were injected with SNU-1 cells (inhibitors NC group or miR inhibitors group). The ordinate indicates the number of nodules per section.
